# A multimodal ensemble stacking model improves brain age prediction and reveals associations with schizophrenia symptoms

**DOI:** 10.3389/fpsyt.2025.1600479

**Published:** 2025-09-04

**Authors:** Junhyeok Lee, Seo Yeong Kim, Hye Won Park, Juhyuk Han, Sung Woo Joo, Jungsun Lee, Won Hee Lee

**Affiliations:** ^1^ Department of Software Convergence, Kyung Hee University, Yongin, Republic of Korea; ^2^ Department of Artificial Intelligence, Kyung Hee University, Yongin, Republic of Korea; ^3^ Department of Psychiatry, Asan Medical Center, University of Ulsan College of Medicine, Seoul, Republic of Korea

**Keywords:** machine learning, schizophrenia, brain age, magnetic resonance imaging, multimodal neuroimaging data

## Abstract

**Introduction:**

Brain age prediction using neuroimaging and machine learning has emerged as a promising approach to assess brain health and detect deviations associated with neurological and psychiatric disorders. The difference between chronological age and predicted brain age, known as brain-predicted age difference (brainPAD), is considered a potential biomarker for advanced brain aging. However, most studies rely on single-modality imaging, limiting predictive accuracy and generalization. This study aimed to enhance brain age prediction by integrating multimodal neuroimaging—structural MRI (sMRI) and diffusion MRI-derived fractional anisotropy (FA)—and evaluating its effectiveness in both healthy individuals and schizophrenia patients.

**Methods:**

We analyzed a large, multi-site dataset of 2,558 healthy individuals (aged 12–88 years) using machine learning approaches to assess the impact of multimodal inputs on brain age prediction. A stacking model combining sMRI and FA features was developed and validated. To evaluate cross-dataset generalization, the model was tested on an independent dataset comprising 56 healthy individuals (aged 20–58 years) and 48 schizophrenia patients (aged 19–65 years). Statistical analyses were conducted to compare brainPAD scores between groups and assess correlations with clinical measures in schizophrenia patients.

**Results:**

The multimodal stacking model achieved superior prediction performance compared to single-modality models, with a mean absolute error (MAE) of 2.675 years and Pearson’s correlation (r) of 0.970 between predicted and chronological age in the internal test set. External validation on the COBRE dataset demonstrated MAE of 4.556 years (r = 0.877) for healthy controls and 6.189 years (r = 0.873) for patients with schizophrenia. Schizophrenia patients exhibited significantly higher brainPAD scores compared to healthy controls (t = 3.857; *p* < 0.001; Cohen’s d = 0.769), suggesting advanced brain aging. Additionally, brainPAD was significantly correlated with symptom severity scores in schizophrenia (ρ = 0.331–0.337, *p* < 0.05).

**Discussion:**

Our findings demonstrate that integrating sMRI and FA features improves brain age prediction accuracy and generalization. Furthermore, the correlation between brainPAD and clinical symptoms highlights its potential as a biomarker for disease progression and treatment monitoring. These results underscore the value of multimodal neuroimaging and machine learning in advancing psychiatric neuroimaging research and paving the way for clinical applications in schizophrenia and related disorders. Further investigation with larger sample sizes is required to validate and extend these findings.

## 1 Introduction

Schizophrenia is a severe and chronic mental disorder characterized by a constellation of positive (e.g., hallucinations, delusions) and negative (e.g., social withdrawal, blunted affect) symptoms, and cognitive impairments that significantly impact daily functioning and quality of life (1–3). Despite decades of research, the underlying neurobiological mechanisms driving the development and progression of schizophrenia remain incompletely understood. This lack of clear biological markers has hindered efforts toward early detection, accurate diagnosis, and targeted treatment interventions.

Emerging evidence suggests that individuals with schizophrenia exhibit advanced brain aging compared to healthy controls (4–8). The concept of “brain age” refers to a measure derived from neuroimaging data that estimates the biological age of an individual’s brain (9). While healthy brain aging is characterized by gradual structural and functional changes, schizophrenia patients tend to exhibit neuroanatomical and connectivity patterns more akin to those of older individuals, even from the early stages of the disorder (10, 11). Brain age prediction using machine learning techniques applied to neuroimaging data, such as structural magnetic resonance imaging (sMRI) and diffusion MRI (dMRI), has shown promise in capturing this advanced brain aging trajectory in schizophrenia (7, 8). The discrepancy between an individual’s predicted brain age and their chronological age, termed the “brainPAD”, has emerged as a potential transdiagnostic biomarker reflecting advanced brain aging observed multiple psychiatry disorders, including schizophrenia, bipolar disorder, and other neuropsychiatric disorders (4, 5, 7, 12).

Elucidating the relationship between brain age and the clinical manifestations of schizophrenia could provide valuable insights into the underlying neurobiology and pathophysiological processes involved (7, 8) Moreover, brain age prediction models may aid in the development of objective diagnostic tools and facilitate early intervention strategies, ultimately improving treatment outcomes and quality of life for individuals with schizophrenia (4, 13, 14). Correlations between brainPAD and symptom severity, cognitive deficits, and functional impairment have been reported, suggesting that brainPAD could serve as a potential biomarker of overall disease burden (10, 11). For instance, a larger brainPAD in schizophrenia is associated with greater cognitive impairments and deficits (6, 10, 15). In first-episode schizophrenia patients, a reduction in brainPAD was observed after a few months of antipsychotic treatment, indicating brain age may track treatment-related improvements in brain integrity and cognitive functioning (10). Among unaffected relatives of schizophrenia patients, a larger brainPAD was associated with lower cognitive performance, suggesting advanced brain aging may relate to genetic liability for cognitive deficits in psychosis (15). Multiple studies converge on the finding that an increased brainPAD, reflecting advanced brain aging, is associated with more severe cognitive impairments in schizophrenia (7).

Several studies have investigated the accuracy of brain age prediction in schizophrenia using multimodal neuroimaging data. While it is well established that combining multiple modalities improves age prediction accuracy compared to single-modality models (13, 14, 16, 17), recent discussions in the literature emphasize that predictive accuracy alone is not sufficient to establish the utility of a brain age model in clinical populations (18). In the context of schizophrenia, the key question is whether multimodal models demonstrate greater sensitivity (or utility) in capturing individual differences related to disease severity, symptom profiles, or functional outcomes, beyond what is achievable with single-modal approaches (18–21). As highlighted by Jirsaraie et al. (19) in their systematic review, enhanced prediction accuracy from multimodal integration is expected; however, whether such models yield stronger associations with clinically meaningful phenotypic variation remains an important open question that requires direct investigation.

Our multimodal approach incorporates FA maps derived from dMRI alongside sMRI data based on substantial evidence of white matter abnormalities in schizophrenia (22–26). Previous research has demonstrated altered white matter integrity in schizophrenia, with FA reductions observed across multiple white matter tracts (22). By incorporating this schizophrenia-relevant modality, our model is designed to capture neuroanatomical variations potentially more sensitive to the disease processes.

Building on prior research demonstrating the advantages of multimodal neuroimaging for brain age prediction, the present study aimed to (i) replicate previous findings that combining structural and diffusion MRI features improves prediction accuracy in a large healthy control cohort, and (ii) apply the resulting multimodal models to a schizophrenia (SZ) cohort to investigate potential clinical relevance. Specifically, we assessed whether integrating T1-weighted structural MRI (sMRI) and fractional anisotropy (FA) maps from diffusion MRI (dMRI) enhances brain age prediction accuracy relative to single-modality models. Furthermore, we examined whether the brain-predicted age difference (brainPAD) is associated with clinical symptom severity, as measured by the Positive and Negative Syndrome Scale (PANSS) (27, 28), in individuals with SZ (29).

We used sMRI and dMRI data from 2,558 healthy participants (n = 2,558, female: 1,327; age range: 12–88 years) to train and evaluate five representative machine learning models: support vector regression (30), relevance vector regression (31), least absolute shrinkage and selection operator (Lasso) regression (32), Gaussian process regression (33), and random forest regression (34). Feature matrices derived from preprocessed MRI scans were standardized and reduced in dimensionality using principal component analysis (PCA) (35). After validating model performance in healthy controls, we applied the best-performing single-modal and multimodal models to a schizophrenia cohort (n = 48) and assessed the relationship between brainPAD and clinical symptoms.

## 2 Materials and methods

**Figure 1:**
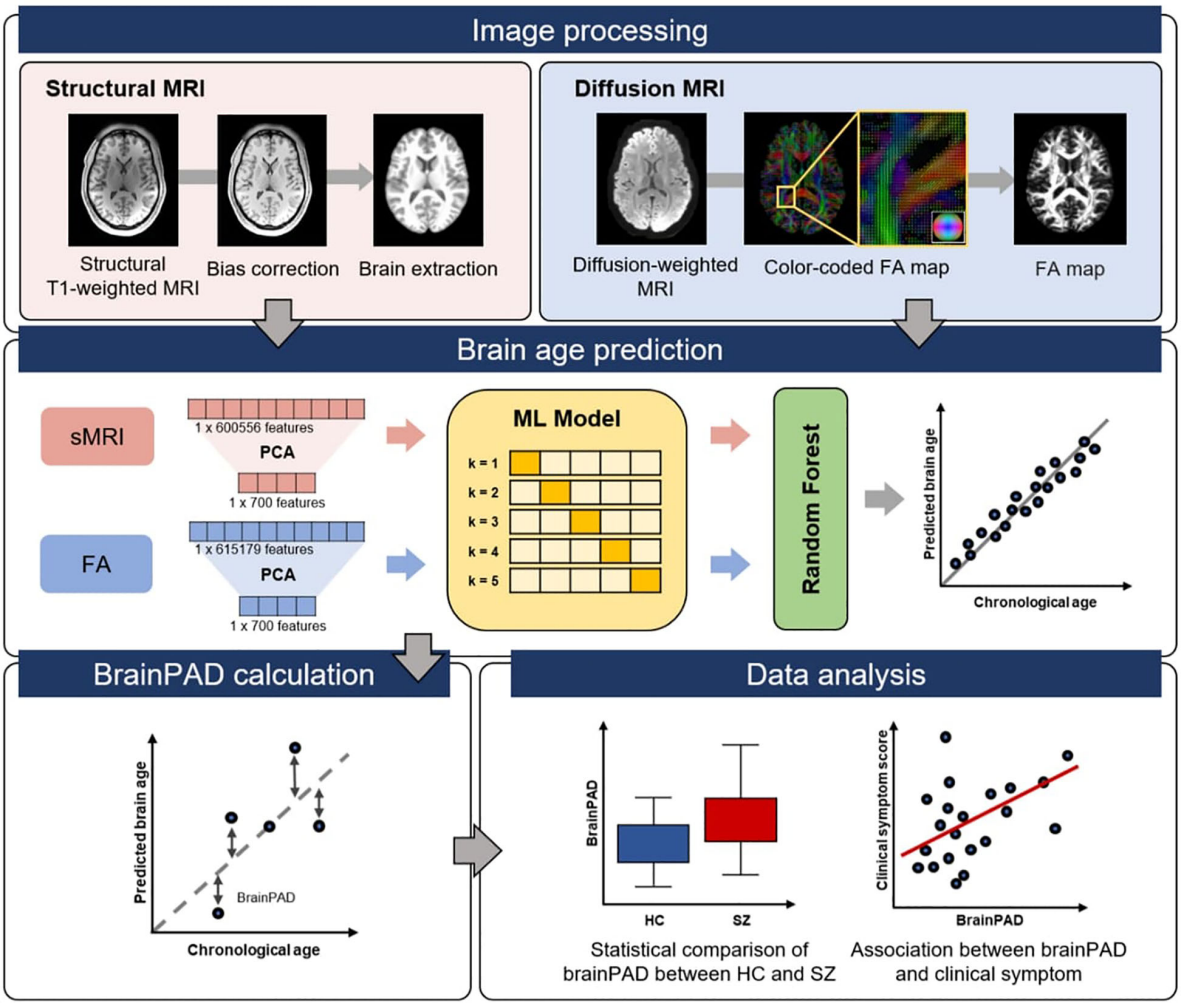
Overview of the workflow for brain age prediction using multimodal neuroimaging data.

An overview of the workflow for brain age prediction using multimodal neuroimaging data is shown in **Figure 1**.

### 2.1 Datasets

We employed structural, T1-weighted magnetic resonance imaging (sMRI) and diffusion magnetic resonance imaging (dMRI) data from a large, multi-site cohort of 2,558 healthy participants (1,327 females, age range 12–88 years) for the training dataset for our brain age prediction model. This data originated from established studies including the Human Connectome Project (HCP) S1200 release (n = 1,060, 574 females, age range 22–37 years) (36), the Cambridge Center for Ageing and Neuroscience (Cam-CAN; n = 638, 321 females, age range 18–88 years) (37), the Southwest University Longitudinal Imaging Multimodal Brain Data Repository (SLIM; n = 550, 298 females, age range 17–27 years) (38), and the Consortium for Reliability and Reproducibility study (CoRR; n = 310, 134 females, age range 12–62 years) (39). All subjects were confirmed to have no neurological or psychiatric history and were cognitively healthy. To assess the generalization performance and utility of the trained models, we utilized sMRI and dMRI data from two separate groups: 56 healthy controls (HC) with no history of major psychiatric disorders (15 females, age range 20–58 years) and 48 schizophrenia patients meeting the DSM-IV criteria (12 females, age range 19–65 years) (40). Both datasets were provided by the Center of Biomedical Research Excellence (COBRE) (40). While the original COBRE dataset included approximately 100 HC and 100 SZ participants, the reduced sample size in this study resulted from the exclusion of participants with inadequate image quality, missing diffusion MRI data, or incomplete clinical measures. We used deidentified data from publicly available repositories. Ethical approvals and informed consents were obtained locally for each study, covering both participation and subsequent data sharing. Sample characteristics of the datasets used in this study are shown in **Table 1** (40).

**Table 1:** Demographics of datasets used in this study.

Dataset	Group	N	Male/Female	Age range (Mean ± SD)
HCP	HC	1,060	486/574	22-37 (28.7 ± 3.7)
Cam-CAN	HC	638	317/321	18-88 (54.5 ± 18.6)
SLIM	HC	550	252/298	17-27 (20.0 ± 1.3)
CoRR	HC	310	176/134	12-62 (22.5 ± 7.8)
COBRE	HC	56	41/15	20-58 (37.4 ± 11.4)
	SZ	48	36/12	19-65 (37.2 ± 13.5)

### 2.2 Data processing

Structural T1-weighted images were preprocessed using the Computational Anatomy Toolbox 12 (CAT12) for each subject with default settings (41). Skull-stripping was performed to remove non-brain tissues from the images, enhancing the focus on brain structures. Intensity inhomogeneities due to magnetic field variations were corrected to improve image quality and uniformity. This correction was performed using the spatially adaptive non-local means (SANLM) denoising filter. The preprocessed images were normalized to the Montreal Neurological Institute (MNI) space using DARTEL (Diffeomorphic Anatomical Registration using Exponentiated Lie algebra) for inter-subject alignment. The normalization process included high-dimensional warping to match the MNI template, ensuring precise anatomical correspondence across subjects. The resulting preprocessed sMRI images had a voxel size of 113×137×113 and isotropic spatial resolution of 1.5 mm³. A quality check was performed on the preprocessed images using the automated quality control measures provided by CAT12, which include checking for artifacts, homogeneity, and image resolution.

Diffusion-weighted MRI images were preprocessed using a combination of DSI-Studio (42) and FSL (FMRIB Software Library, version 6.0, https://fsl.fmrib.ox.ac.uk/fsl/fslwiki) to reconstruct diffusion tensor images (DTI) (14, 43). Eddy current-induced distortions and subject motion were corrected using the *eddy* tool in FSL. This step aligned all the diffusion-weighted volumes to a common reference, correcting for motion and eddy current-induced distortions. A brain mask was generated from the b_0_ image using the Brain Extraction Tool (BET) in FSL to exclude non-brain tissues. Susceptibility-induced distortions were corrected using the FSL tool *topup*, which requires an additional field map or phase-encoding reversed images if available. Fractional anisotropy (FA) was computed using the *dtifit* tool in FSL. FA measures directional diffusion, ranging from zero (isotropic) to one (anisotropic), and is a commonly used quantitative dMRI metric in the literature (33, 34). The FA map was then co-registered to the corresponding structural MRI (sMRI) data using affine transformation followed by symmetric normalization (SyN) diffeomorphic transformation for optimal spatial alignment as implemented in the Advanced Normalization Tools (ANTs) (36, 44). This ensured that the FA map had the same voxel size (113×137×113 with 1.5 mm isotropic voxels) and spatial orientation as the sMRI data. Quality control for the DTI data was performed following the ENIGMA-DTI protocols (45). This included visual inspection of the FA maps for any artifacts, misregistration, or other anomalies. Moreover, standardized scripts from ENIGMA were used to assess and report on image quality metrics such as signal-to-noise ratio (SNR), outliers, and other relevant QC measures (https://enigma.ini.usc.edu/protocols/dti-protocols/).

Both the preprocessed sMRI and FA images were masked using the MNI152 template and the FMRIB58 FA template masks, respectively. The masked images were then vectorized (converted into a single feature vector) and subsequently normalized using z-scores. This resulted in the creation of individual feature matrices for each subject. Each feature matrix included 600,556 features from sMRI data and 615,179 features from FA data.

### 2.3 Principal component analysis

We employed principal component analysis (PCA) with singular value decomposition (SVD) (35) to reduce the dimensionality of the feature matrix. PCA is a linear dimensionality reduction technique that transforms high-dimensional data into a lower-dimensional space, capturing the maximal variance in the data. We applied PCA to the feature matrix before modeling, resulting in reduced dimensional data derived from the original neuroimaging features. The selection of principal components for retention was based on the explained variance ratio in each model. In all models, including single-modality models using only sMRI or FA data and the multimodal model combining sMRI and FA data, we retained the top 700 principal components, which explained 60% of the total variance observed in the dataset.

To assess the impact of dimensionality on prediction accuracy, we further evaluated the influence of the number of retained principal components on brain age prediction performance for each model. We tested different scenarios by retaining components representing 20%, 40%, 60%, 80%, and 90% of the total variance. This allowed us to examine how the number of principal components retained affects the accuracy of brain age prediction for each model.

### 2.4 Machine learning algorithms

We utilized five widely used machine learning algorithms to capture potential linear and non-linear relationships between multimodal neuroimaging features (sMRI and FA) and brain age (46–54). The linear regression models included support vector regression (SVR) (30), relevance vector regression (RVR) (31), and least absolute shrinkage and selection operator (Lasso) regression (4, 32, 51, 53, 55). For non-linear modeling, we used Gaussian process regression (GPR) (33) and random forest (RF) regression (34). This selection of models allows us to investigate the influence of both linear and non-linear relationships on the prediction of brain age. Detailed descriptions of each method are provided in the **Supplementary Materials**.

### 2.5 Brain age prediction

The data was randomly divided into a training set (80%) and an internal test set (20%) to evaluate the model’s generalization to unseen data. To mitigate potential bias due to age and gender differences, we ensured statistically similar distributions of these variables in both sets. To evaluate the model’s generalization to unseen data, the dataset was randomly divided into a training set (80%) and an internal test set (20%) using stratified sampling to preserve the distributions of scanner site, sex, and age. To statistically verify this balance, we conducted a Mann-Whitney U test for age, which revealed no significant difference between the training and test sets (U = 520570.5, *p* = 0.790). For categorical variables, chi-square tests confirmed that the distributions of sex ( $\chi^{2}$ = 0, p = 1.0) and scanner site ( $\chi^{2}$ = 0.23, *p* = 0.972) were not significantly different across the two sets. These results indicate that demographic and site-related confounds were adequately controlled in the dataset split. Prior to model training, sMRI and FA features were standardized using the robust scaler from scikit-learn (56), which centers data around the median and scales it by the interquartile range (IQR) to improve model performance by reducing the influence of outliers.

For single-modality brain age prediction, we explored five widely-used regression algorithms with strengths in handling different data characteristics: SVR (30) and RVR (31) for handling high-dimensional data with potential sparsity, Lasso regression (32) for feature selection and interpretability, GPR (33) for non-parametric modeling of nonlinear relationships, and RF for robustness to noise and overfitting. We trained separate models for both sMRI and FA data using these algorithms.

For multimodal brain age prediction, we employed a stacking framework to integrate information across modalities. This approach aligns with recent studies that utilized model stacking to combine features from different imaging modalities (57, 58). Initially, single-modality models were trained using k-fold cross-validation on the training set, generating brain age predictions for each subject based on sMRI and FA features separately. These predictions served as input features for a second-level model (i.e., an RF regressor) that was trained to predict chronological age based on the combined single-modality outputs. The same training/testing splits used in the first-level models were maintained to ensure that the stacked model was evaluated only on unseen internal test data. The second-level RF model was optimized via grid search over two hyperparameters: the number of trees (10, 50, or 100) and maximum tree depth (5, 10, 20, or None, where None allows trees to grow to full depth). Model performance was evaluated using 5-fold cross-validation within the training set for hyperparameter tuning and subsequently assessed on the internal test set using two key metrics: mean absolute error (MAE), which quantifies the average absolute difference between predicted and actual age, and Pearson’s correlation coefficient (r), which measures the linear association between predicted and chronological age.

Finally, the trained model was applied to an external validation set (i.e., the COBRE dataset), which includes both healthy controls and patients with schizophrenia. This allowed us to assess the generalization of brain age predictions and to examine associations between brainPAD and clinical symptom severity in patients with schizophrenia.

### 2.6 Age-bias correction

BrainPAD was computed by subtracting an individual’s chronological age from their predicted brain age. Positive brainPAD values indicate a predicted brain age that is older than the individual’s chronological age, while negative brainPAD values suggest a predicted brain age that is younger. Due to the statistical phenomenon of regression to the mean in regression analysis (59, 60), brainPAD values tend to be overestimated in younger individuals and underestimated in older individuals. To address this age-related bias in brain age prediction, we applied the age-level correction method proposed by Zhang et al. (61). This method involves fitting a linear regression model between brainPAD values and chronological age using healthy control (HC) data from the COBRE sample. The fitted model estimates the expected brainPAD for each age, which is then subtracted from the original brainPAD scores to yield age-corrected estimates. Unlike traditional sample-level correction approaches (60) that adjust only the overall mean brainPAD to zero, Zhang et al.’s method corrects residual linear biases at each individual age level. This ensures effective bias removal across the entire age spectrum, providing unbiased brainPAD estimates regardless of chronological age (61). For samples of age a, the age-bias corrected brainPAD, $brainPAD_{i}^{ac}$ , was computed as follows:


$$brainPAD_{i}^{ac}=\frac{brainPAD_{i}-\;\mu_{a}}{\sigma_{a}}$$

where $\mu_{a}$ and $\sigma_{a}$ denote the mean and standard deviation of brainPAD over samples of age *a*, respectively. This correction can eliminate the bias as it ensures that the mean of $brainPAD_{i}^{ac}$ of the same age a is zero (61):


$${Ε}_{a}\left[brainPAD_{i}^{ac}\right]=\;{Ε}_{a}\left[brainPAD_{i}-\mu_{a})/\sigma_{a}\right]=\frac{{Ε}_{a}\left[brainPAD_{i}\right]-\mu_{a}}{\sigma_{a}}=0$$

### 2.7 Statistical analysis

Between-group differences in brainPAD were assessed using a multiple regression model with age, sex, and diagnostic group (SZ vs. HC) as covariates. This approach allows for the isolation of diagnosis-specific effects on brain aging while controlling for demographic variables that are known to influence brain structure and brain age prediction accuracy. Effect sizes were quantified using Cohen’s d, calculated directly from the regression model’s t-statistics to provide a standardized measure of the magnitude of group differences (63).

The COBRE sample provided a unique opportunity to explore the functional significance of brain age prediction in schizophrenia (40). This dataset includes MRI data from schizophrenia patients along with clinician-collected symptom severity scores using the Positive and Negative Syndrome Scale (PANSS) (28). The PANSS provides comprehensive assessment of schizophrenia symptoms, including total scores and subscale scores for positive symptoms, negative symptoms, and general psychopathology.

Prior to correlation analysis, Shapiro-Wilk normality tests were performed to assess the distribution of all variables (**Supplementary Table S1**). Based on these results, Spearman’s rank correlation coefficient was used to assess these non-parametric associations while controlling for chronological age and sex. A supplementary analysis using Pearson’s correlation is provided in the supplement (**Supplementary Table S2**). To account for multiple comparisons, we employed false discovery rate (FDR) correction with a *q*-value threshold of 0.05 (62). This approach helps to control potential inflation of Type I errors arising from conducting numerous statistical tests. All statistical analyses were performed using Python, v. 3.8.16 with the statsmodels, v. 0.14.1 package.

## 3 Results

### 3.1 Brain age prediction performance

**Figure 2:**
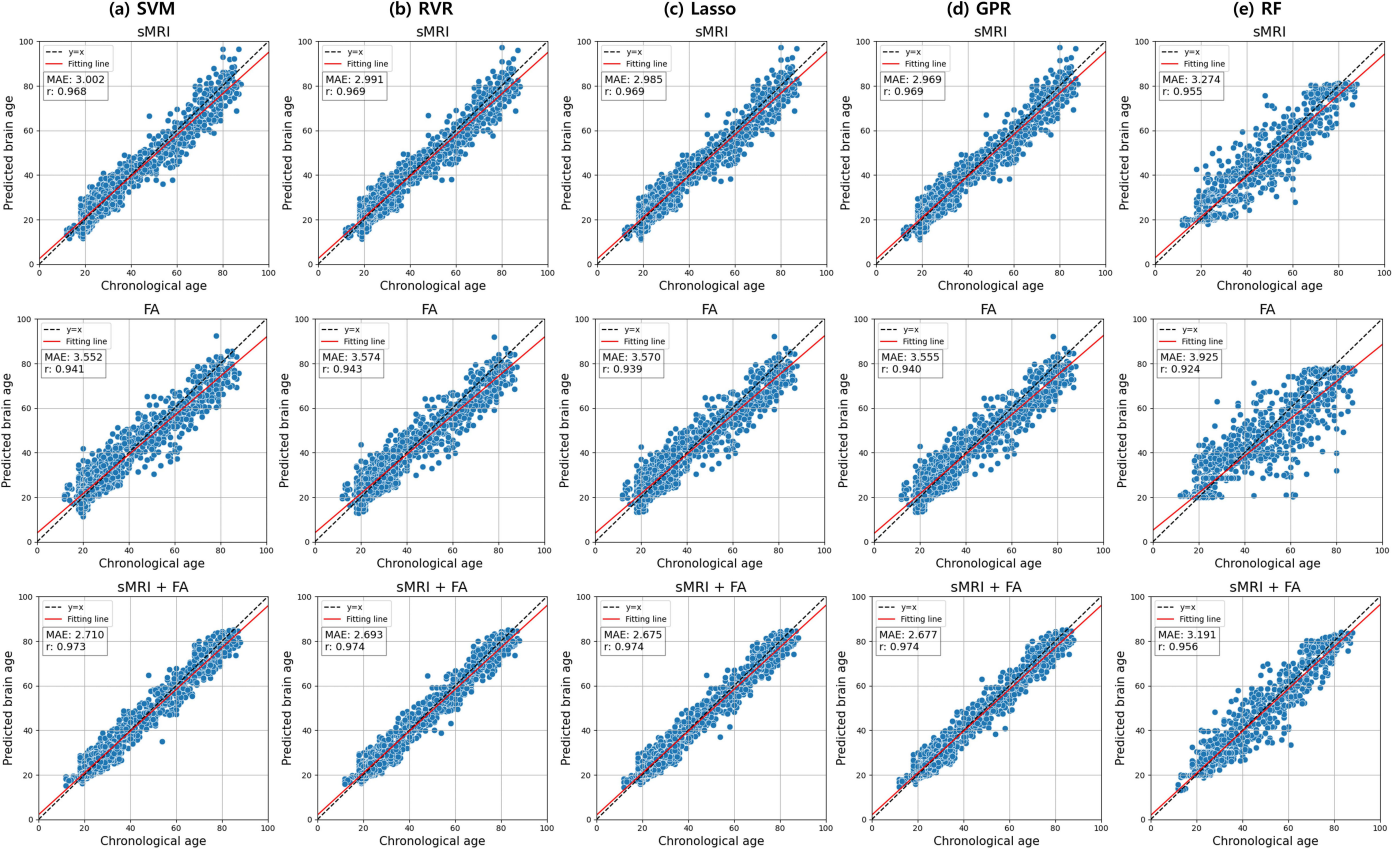
Scatter plots showing pairwise correlations between chronological age and predicted brain age for five machine learning algorithms applied to single-modality (sMRI and FA) and multimodal (sMRI+FA) data: **(a)** support vector regression (SVR), **(b)** relevance vector regression (RVR), **(c)** least absolute shrinkage and selection operator (Lasso) regression, **(d)** Gaussian process regression (GPR), and **(e)** random forest (RF) regression. Pearson correlation coefficient (R) and mean absolute error (MAE) are shown for each association.

**Table 2** presents the performance metrics of various machine learning models for brain age prediction. The multimodal stacking model combining sMRI and FA features with Lasso regression demonstrated the lowest prediction error (MAE = 2.675, r = 0.974) (**Figure 2**). sMRI-only models yielded MAE values ranging from 2.969 to 3.274, while FA-based models produced MAE values between 3.552 and 3.925. The multimodal models that incorporated both sMRI and FA data achieved MAE values ranging from 2.675 to 3.191. Among the evaluated approaches, the stacking method with Lasso regularization produced the most accurate brain age predictions when combining multimodal neuroimaging features.

**Table 2:** Performance comparison of different machine learning algorithms applied to single-modality (sMRI and FA) and multimodal (sMRI+FA) neuroimaging data.

Model		Data	sMRI		FA		sMRI+FA	
			MAE	r	MAE	r	MAE	r
Linear Model	Support Vector Regression	Train	1.848	0.983	2.171	0.976	1.622	0.989
		Test	3.002	0.968	3.552	0.941	2.710	0.973
	Relevance Vector Regression	Train	2.097	0.986	2.497	0.980	1.702	0.990
		Test	2.991	0.969	3.574	0.943	2.693	0.974
	Lasso Regression	Train	2.050	0.986	2.425	0.980	1.695	0.990
		Test	2.985	0.969	3.570	0.939	**2.675**	**0.974**
Non-Linear Model	Gaussian Process Regression	Train	2.045	0.986	2.422	0.981	1.675	0.990
		Test	2.969	0.969	3.555	0.940	2.677	0.974
	Random Forest Regression	Train	2.768	0.972	3.263	0.960	2.271	0.981
		Test	3.274	0.955	3.925	0.924	3.191	0.956

The best and second-best results are marked in bold and underline on the unseen test set, respectively.

**Figure 3:**
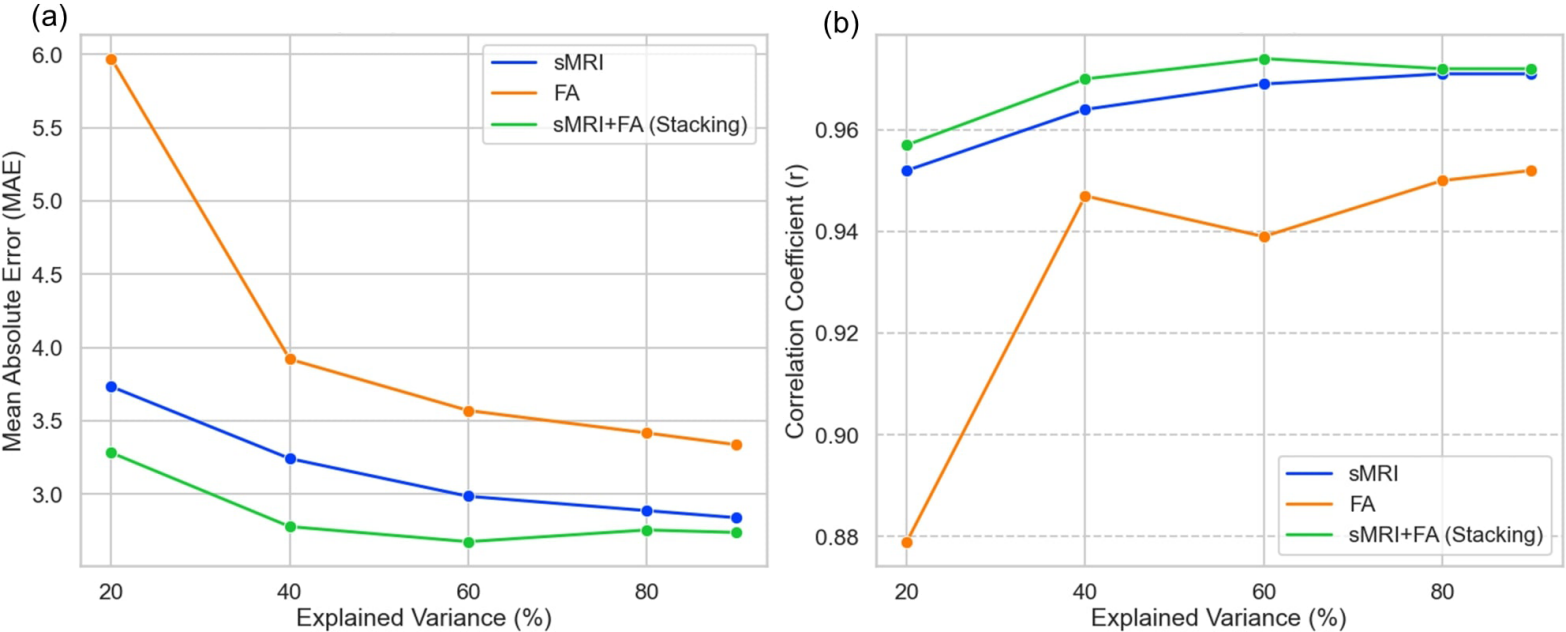
**(a)** Mean absolute errors (MAE) and **(b)** correlation coefficients between predicted brain age and chronological age in singlemodality (sMRI and FA) and multimodal (sMRI+FA) models.

We examined the relationship between the number of principal components (PCs) retained from PCA and brain age prediction performance using the best-performing models. Models were evaluated using PCs that explained 20%, 40%, 60%, 80%, and 90% of the total variance in the original feature set. Prediction performance varied with the amount of variance retained, with the lowest MAE observed when approximately 60% of the variance was preserved (**Table 3**, **Figure 3**). Performance remained stable or slightly decreased when additional components beyond this point were included. These results show that retaining 60% of the variance provided optimal prediction accuracy in this dataset.

**Table 3:** Comparison of predictive performance in brain age prediction by the number of PCA components retained on the internal test set.

Explained variance	Data	sMRI		FA		sMRI+FA	
		MAE	r	MAE	r	MAE	r
20%	Train	3.632	0.956	5.687	0.894	2.859	0.972
	Test	3.733	0.952	5.971	0.879	3.282	0.957
40%	Train	2.673	0.977	3.090	0.969	2.146	0.984
	Test	3.242	0.964	3.920	0.947	2.778	0.970
60%	Train	2.050	0.986	2.425	0.980	1.695	0.990
	Test	2.985	0.969	3.570	0.939	**2.675**	**0.974**
80%	Train	1.469	0.992	1.564	0.991	1.178	0.995
	Test	2.887	0.971	3.417	0.950	2.755	0.972
90%	Train	1.059	0.996	1.101	0.995	0.837	0.997
	Test	2.839	0.971	3.337	0.952	2.738	0.972

The best results are marked in bold.

Notably, the stacking model that combined both sMRI and FA features consistently achieved the best brain age prediction accuracy across all tested proportions of explained variance (**Table 3**). **Figure 3** displays the performance metrics (MAE and correlation) for each model configuration based on the proportion of variance explained by the PCs. The multimodal stacking model maintained superior performance (lowest MAE, highest correlation) regardless of the specific variance threshold, demonstrating its robustness to dimensionality reduction parameters. This consistent performance advantage suggests that integrating complementary information from both structural and diffusion imaging provides more stable and accurate brain age predictions than either modality alone, even under varying feature selection conditions.

### 3.2 Generalization performance and utility in the COBRE sample

We evaluated the generalization of the best-performing multimodal stacking model by testing it on independent test sets within the COBRE sample. This involved testing the model on new data that wasn’t used for training to evaluate its ability to predict brain age in unseen subjects. The multimodal model maintained its superior performance, achieving the lowest MAE compared to single-modality models in the COBRE sample (**Table 4**). These results suggest that the multimodal model generalizes well for brain age prediction on unseen data. The multimodal model achieved an MAE of 4.556 and a correlation of 0.877 for healthy controls (HC) and an MAE of 6.189 and a correlation of 0.873 for schizophrenia (SZ) patients in the COBRE sample (**Table 4**).

**Table 4:** Generalization performance of brain age prediction models using single-modality (sMRI or FA) and multimodal (sMRI+FA) data in the COBRE sample.

Group	sMRI		FA		sMRI+FA	
	MAE	r	MAE	r	MAE	r
HC	5.038	0.879	7.509	0.695	**4.556**	0.877
SZ	6.558	0.886	7.489	0.729	**6.189**	0.873

The best results are marked in bold.

**Figure 4:**
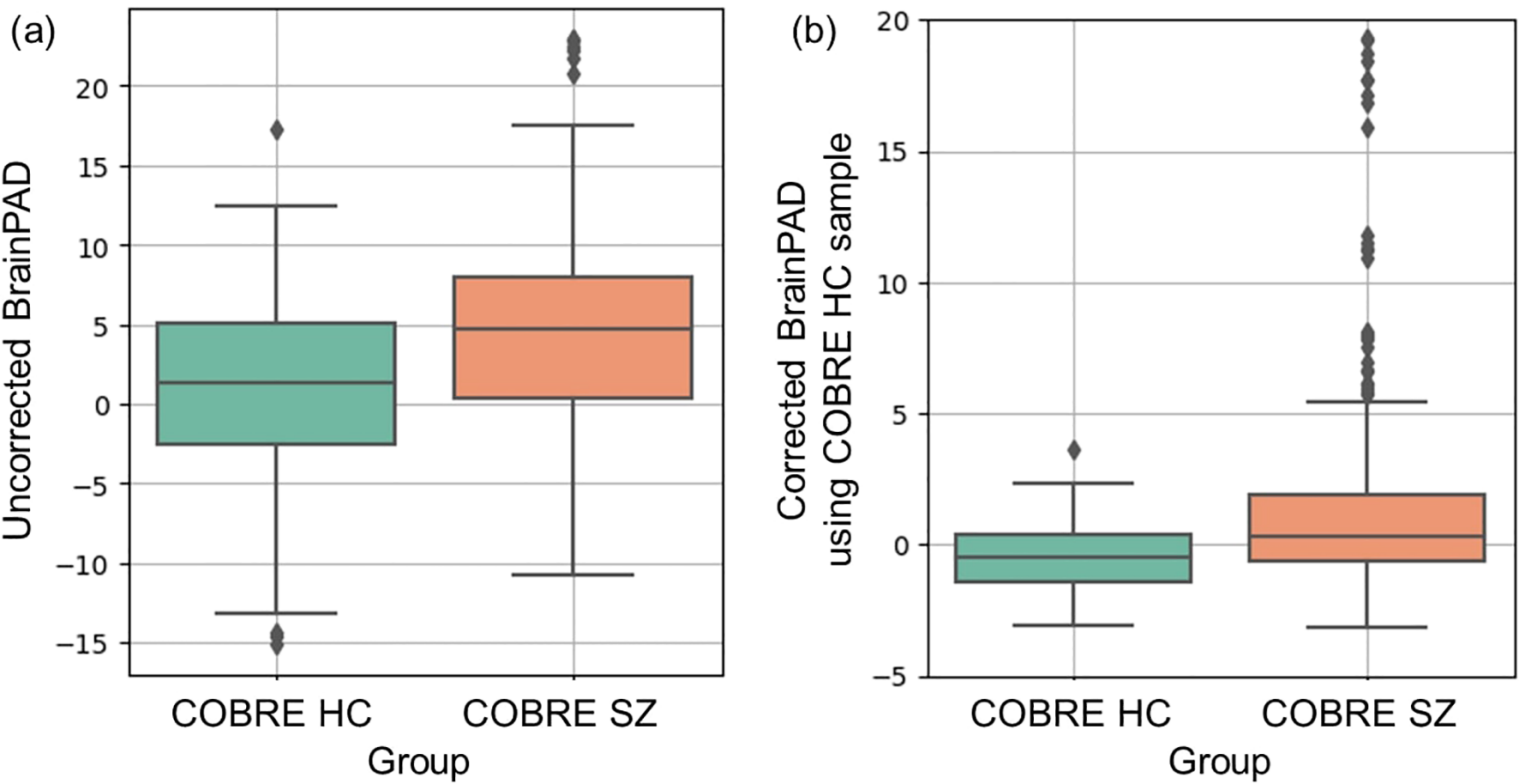
Boxplots of the brain-predicted age difference (brainPAD) for HC and SZ in the COBRE sample: **(a)** Uncorrected brainPAD for HC and SZ. **(b)** Age-corrected brainPAD for HC and SZ using predictions from the COBRE HC group.

We then computed brainPAD for each participant in the COBRE sample (**Figure 4a**). To address potential age-bias, we applied age-bias correction method using brain age predictions from the HC group within COBRE. **Table 5** presents mean brainPAD differences and group comparison statistics for HC versus SZ across three imaging modalities. SZ patients consistently demonstrated elevated brainPAD values relative to HC across all modalities, with the multimodal model showing the strongest group differences (t = 3.857; *p* < 0.001; Cohen’s d = 0.769) (**Figure 4b**). Alternative age-bias correction results based on the training sample are provided in the **Supplementary Materials** (**Supplementary Figure S1**; **Supplementary Table S3**).

**Table 5:** Comparison of brainPAD values between healthy controls (HC) and patients with schizophrenia (SZ) for both uncorrected and age-bias corrected estimates.

Modality	Metric	Uncorrected brainPAD		Corrected brainPAD	
		HC	SZ	HC	SZ
sMRI	Mean (SD)	1.298 (5.813)	4.340 (6.568)	-0.614 (1.378)	1.624 (4.210)
	*b* (SE)	2.995 (0.825)		2.242 (0.605)	
	*t*	3.629		3.702	
	*p*	< 0.001		< 0.001	
	*d*	0.728		0.739	
FA	Mean (SD)	-4.774 (8.357)	-2.479 (9.289)	-0.790 (1.252)	1.690 (6.872)
	*b* (SE)	2.205 (1.006)		2.490 (0.948)	
	*t*	2.190		2.624	
	*p*	0.030		0.010	
	*d*	0.439		0.523	
sMRI + FA	Mean (SD)	0.310 (5.626)	3.938 (6.597)	-0.523 (1.322)	1.687 (3.988)
	*b* (SE)	3.584 (0.939)		2.215 (0.574)	
	*t*	3.814		3.857	
	*p*	< 0.001		< 0.001	
	*d*	0.765		0.769	

Group means and standard deviations (SD) are provided. Multiple regression models were used to adjust for chronological age and sex. The regression coefficient *b* with standard error (SE) represents the unstandardized effect size (in years) for group differences estimated from the multiple regression model. Cohen’s d values represent the effect sizes for group differences.

**Figure 5:**
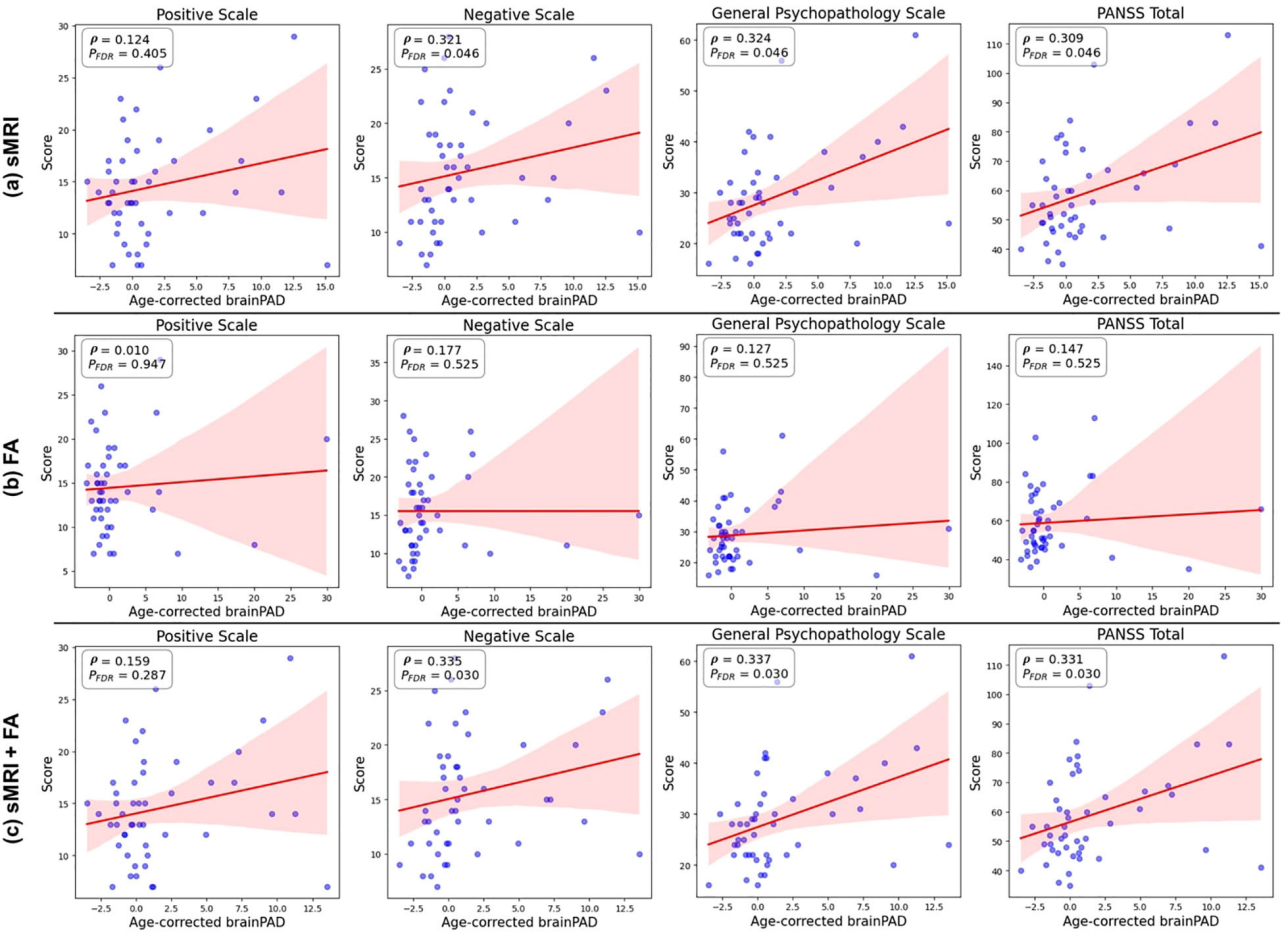
Associations between age-corrected brainPAD and PANSS scores. Scatter plots showing the correlations between age-corrected brainPAD estimated from the Lasso regression models and each of the four PANSS symptom scores (positive, negative, general psychopathology, and total) in patients with schizophrenia, controlling for chronological age and sex. The models are based on **(a)** structural MRI (sMRI) data only, **(b)** fractional anisotropy (FA) data only, and **(c)** combined sMRI and FA data (stacking model). Higher brainPAD scores indicate greater predicted age difference compared to chronological age.

**Figure 5** shows the association between age-corrected brainPAD values and PANSS symptom scores (total and three subscale scores) in individuals with schizophrenia. Significant positive correlations were observed between brainPAD and negative symptoms (ρ = 0.335, uncorrected p = 0.021, FDR-corrected p = 0.030), general psychopathology symptoms (ρ = 0.337, uncorrected p = 0.020, FDR-corrected p = 0.030), and overall PANSS total scores (ρ = 0.331, uncorrected p = 0.022, FDR-corrected p = 0.030). In contrast, the correlation between brainPAD and positive symptoms was not statistically significant (ρ = 0.159, uncorrected p = 0.286, FDR-corrected p = 0.287). Both the multimodal and sMRI-based models demonstrated comparable correlation patterns with negative and general psychopathology symptoms, as visualized in **Figure 5**.

## 4 Discussion

This study investigated the efficacy of multimodal neuroimaging data (sMRI and FA) in predicting brain age using machine learning algorithms. We compared the performance of various models and explored the relationship between predicted brain age and clinical symptoms in schizophrenia. Our findings highlight the potential of multimodal brain imaging for improved brain age prediction and its association with clinical manifestations.

### 4.1 Multimodal advantage for brain age prediction

Our findings support the use of multimodal data (sMRI and FA) for brain age prediction. Our results show that the multimodal stacking model with Lasso regression achieved superior performance compared to single-modality models, highlighting the value of combining these modalities for brain age prediction. Structural MRI provides detailed anatomical information about gray and white matter distribution (64, 65), while FA offers insights into white matter microstructure and the integrity of connecting pathways within the brain (22). Combining these complementary pieces of information likely leads to a more comprehensive representation of the brain and its age-related changes. Furthermore, these results support the notion that an integrative approach leveraging multiple data sources can enhance the precision and accuracy of brain age predictions (13, 51, 54, 58), which is crucial for identifying deviations linked to neuropsychiatric disorders (4, 5, 7, 8). These findings encourage the adoption of multimodal neuroimaging in clinical practice, where it could potentially enhance diagnostic accuracy and individualized treatment planning (7, 8).

Relatively few studies have investigated brain age prediction with multimodal brain features. Existing research highlights the potential of combining different modalities. Rokicki et al. examined the performance of brain age prediction models using various combinations of MRI-derived features and cerebral blood flow data, achieving high prediction accuracy (r² = 0.77, MAE = 6.4 years) when integrating all modalities in a healthy population (n = 750, aged 18–86 years) (13). Similarly, Liem et al. reported improved prediction accuracy (MAE = 4.29 years) using multimodal data that included cortical anatomy and whole-brain functional connectivity in a healthy cohort (n = 2,354, aged 18–86 years) (58). Cole explored brain age prediction in a healthy population (n = 2,205, aged 45–80 years) using six imaging modalities (T1-weighted MRI, T2-FLAIR, T2*, diffusion MRI, task functional MRI, and resting-state functional MR), achieving high accuracy with T1-weighted and diffusion MRI (51).

Jirsaraie et al. (19) observed that multimodal brain age models demonstrated the strongest effect sizes for chronic brain disorders, including schizophrenia, with models using larger numbers of features being more effective at detecting group differences in these conditions. Our findings are consistent with this observation, as our multimodal model yielded the largest effect size (Cohen’s d = 0.769) for the group difference between schizophrenia patients and healthy controls, compared to single-modality approaches (sMRI: d = 0.739; FA: d = 0.523). Additionally, our results contribute to the literature by demonstrating that multimodal integration can extend beyond group-level discrimination to capture within-group clinical variation, as evidenced by the associations between brainPAD values and PANSS symptom scores. This suggests that carefully optimized multimodal brain age models may provide clinical utility not only for case-control discrimination but also for characterizing symptom severity within patient populations. We acknowledge, however, that further validation with larger and more diverse clinical samples is needed to confirm these observations.

### 4.2 Generalization and robustness

The multimodal model demonstrated good performance when evaluated on the independent COBRE sample, suggesting potential for application to unseen data from external sources. While this result is encouraging, we acknowledge that true generalization cannot be fully assessed using a single external dataset, especially one with a limited sample size and an age range that does not span the full range used for model training. Therefore, future studies involving diverse, multi-site datasets and broader age distributions are necessary to more rigorously evaluate the model’s generalization across different clinical and demographic populations.

In addition to external validation, we also assessed the robustness of the model with respect to dimensionality reduction using PCA. The model exhibited consistent prediction performance across different PCA settings, particularly when components explaining up to 60% of the variance were retained. This stability suggests that the model is not overly sensitive to the specific number of components selected and can reliably extract relevant information from both sMRI and FA modalities. Such robustness enhances the model’s practical utility, as it indicates resilience to variation in preprocessing choices, thereby reducing the risk of overfitting and improving reliability in applied contexts.

While the multimodal model demonstrated promising performance when applied to an independent subset within the COBRE dataset, it is important to acknowledge that true generalization cannot be fully established based on a single dataset with a limited sample size and age range. Moreover, PCA is a widely used technique to reduce the dimensionality of high-dimensional data such as neuroimaging features (35), the number of retained components can substantially impact model performance. Our results demonstrate that the model’s prediction accuracy varies across PCA settings, with performance improving as more variance is retained, and reaching an optimal point around 60% explained variance. Beyond this point, additional components did not lead to further improvements and sometimes slightly degraded performance. This suggests that the model is robust within a certain range of dimensionality reduction, effectively extracting meaningful information from both sMRI and FA data when an appropriate balance between variance preservation and noise reduction is maintained. Therefore, rather than being uniformly stable across all PCA settings, the model shows reliable performance when the retained components capture sufficient but not excessive variance. This property helps reduce the risk of overfitting and enhances the model’s potential for real-world application. This robustness is encouraging for real-world applications, though further validation is needed across more diverse external datasets, clinical sites, and demographic groups to comprehensively evaluate the model’s generalization and clinical utility.

### 4.3 Brain age acceleration and clinical correlates in schizophrenia

Our study observed significantly higher brainPAD values in schizophrenia patients compared to healthy controls, suggesting an “older than chronological age” brain signature in this population. This finding aligns with previous research that has documented neuroanatomical abnormalities and advanced brain aging in schizophrenia (5, 66–68). Furthermore, the positive correlations between brainPAD and symptom severity (negative symptoms, general psychopathology, and overall PANSS scores) provide evidence for a link between advanced brain aging and increased symptom severity in schizophrenia.

These findings contribute to the heterogenous literature on brainPAD-symptom associations in schizophrenia. While large-scale ENIGMA consortium studies using T1-weighted MRI reported elevated brainPAD in schizophrenia, they found no significant associations with clinical symptom measures (PANSS, SANS, or SAPS scores) (5). Similarly, Joo et al. observed a negative association between brainPAD and Full-scale Intelligence Quotient (FSIQ) in chronic schizophrenia patients, though this correlation did not survive multiple comparison correction (66). In contrast, Kim et al. identified significant associations between brainPAD and positive, negative, and total PANSS symptom scores in schizophrenia patients (67), and Chen et al. found that white matter-derived brainPAD correlated positively with negative symptoms and negatively with FSIQ (68). Our findings align with these latter studies, demonstrating positive correlations between brainPAD and PANSS negative, general psychopathology, and total scores. While the multimodal model produced the strongest correlations overall, the sMRI model also demonstrated comparable brainPAD associations with PANSS negative and general psychopathology symptoms (**Figure 5**), suggesting clinical relevance of sMRI features, even when used in isolation. These findings indicate that structural brain measures may be sufficient for capturing brain age-symptom relationships, while the addition of diffusion tensor imaging primarily enhances age prediction accuracy rather than clinical sensitivity to symptom severity.

These results provide preliminary evidence for brain age prediction as a potential biomarker for disease severity, though validation in larger longitudinal cohorts is needed given our relatively small sample size and cross-sectional design. Future research should investigate whether brainPAD can predict treatment response or track disease progression over time, while exploring the underlying neurobiological mechanisms linking brain-predicted age to symptom severity in schizophrenia.

## 5 Limitations and future directions

While this study provides encouraging results, some limitations need to be addressed in future work. First, the current sample size can be expanded to further improve the generalizability and robustness of the findings. Larger datasets would allow for more robust statistical analyses and potentially reveal even more subtle relationships between brain age and clinical features. Second, validation on entirely independent datasets from different populations is crucial to confirm the generalization of the multimodal brain age prediction model. Generalization across populations is essential for ensuring the model’s applicability in diverse clinical settings. A further limitation of this study is that brain age prediction was based only on two imaging modalities (sMRI and FA). While these modalities capture key aspects of brain structure and white matter integrity, previous studies have demonstrated that incorporating additional neuroimaging features, such as resting-state functional connectivity or cerebral blood flow (16, 69), can enhance prediction accuracy by providing complementary functional or physiological information. Our decision to focus on sMRI and FA was guided by their availability and relevance in clinical and research settings. Future work should explore the integration of a broader range of modalities to further improve the precision and biological interpretability of brain age models. Finally, exploring the clinical utility of brainPAD through longitudinal studies is warranted. Longitudinal studies track participants over time, allowing researchers to investigate how brain age prediction changes with disease progression and treatment. Investigating the relationship between brainPAD and cognitive function could be another avenue for future research. Understanding how brain age prediction relates to cognitive performance in schizophrenia could provide valuable insights into the underlying neurocognitive mechanisms of the disease. By addressing these limitations, future research can solidify the potential of brain age prediction as a valuable tool for understanding and managing brain disorders like schizophrenia.

## 6 Conclusion

This study demonstrated the significant advantages of multimodal neuroimaging data (sMRI and FA) for brain age prediction using machine learning approaches. The multimodal stacking model achieved superior performance and generalization compared to single-modality models. Furthermore, the observed link between brainPAD and symptom severity in schizophrenia patients suggests a potential clinical utility for brain age prediction in disease assessment. Future research with larger samples, external validation, and exploration of clinical utility is warranted to further elucidate the potential of brain age prediction for improving our understanding and treatment of neuropsychiatric disorders like schizophrenia.

## Data Availability

The original contributions presented in the study are included in the article/**Supplementary Material**. Further inquiries can be directed to the corresponding author.

## References

[1] American Psychiatric Association D Association AP. Diagnostic and Statistical Manual of Mental Disorders: Dsm-5. Washington, DC: American psychiatric association (2013).

[2] Del Barrio V. Diagnostic and statistical manual of mental disorders. The curated reference collection in neuroscience and biobehavioral psychology. Am Psychiatr Pub. (2016):10.

[3] Kennedy JL Altar CA Taylor DL Degtiar I Hornberger JC. The social and economic burden of treatment-resistant schizophrenia: A systematic literature review. Int Clin Psychopharmacol. (2014) 29:63–76. doi: 10.1097/YIC.0b013e32836508e6, PMID: 23995856

[4] Lee WH Antoniades M Schnack HG Kahn RS Frangou S. Brain age prediction in schizophrenia: does the choice of machine learning algorithm matter? Psychiatry Research: Neuroimaging. (2021) 310:111270. doi: 10.1016/j.pscychresns.2021.111270, PMID: 33714090 PMC8056405

[5] Constantinides C Han LK Alloza C Antonucci LA Arango C Ayesa-Arriola R. Brain ageing in schizophrenia: evidence from 26 international cohorts via the enigma schizophrenia consortium. Mol Psychiatry. (2023) 28:1201–9. doi: 10.1038/s41380-022-01897-w, PMID: 36494461 PMC10005935

[6] Shahab S Mulsant BH Levesque ML Calarco N Nazeri A Wheeler AL. Brain structure, cognition, and brain age in schizophrenia, bipolar disorder, and healthy controls. Neuropsychopharmacology. (2019) 44:898–906. doi: 10.1038/s41386-018-0298-z, PMID: 30635616 PMC6461913

[7] Seitz-Holland J Haas SS Penzel N Reichenberg A Pasternak O. Brainage, brain health, and mental disorders: A systematic review. Neurosci Biobehav Rev. (2024) 159:105581. doi: 10.1016/j.neubiorev.2024.105581, PMID: 38354871 PMC11119273

[8] Baecker L Garcia-Dias R Vieira S Scarpazza C Mechelli A. Machine learning for brain age prediction: introduction to methods and clinical applications. EBioMedicine. (2021) 72. doi: 10.1016/j.ebiom.2021.103600, PMID: 34614461 PMC8498228

[9] Cole JH Franke K. Predicting age using neuroimaging: innovative brain ageing biomarkers. Trends Neurosci. (2017) 40:681–90. doi: 10.1016/j.tins.2017.10.001, PMID: 29074032

[10] Xi Y-B Wu X-S Cui L-B Bai L-J Gan S-Q Jia X-Y. Neuroimaging-based brain-age prediction of first-episode schizophrenia and the alteration of brain age after early medication. Br J Psychiatry. (2022) 220:339–46. doi: 10.1192/bjp.2021.169, PMID: 35049480

[11] Kim W-S Heo D-W Shen J Tsogt U Odkhuu S Kim S-W. Stage-specific brain aging in first-episode schizophrenia and treatment-resistant schizophrenia. Int J Neuropsychopharmacol. (2023) 26:207–16. doi: 10.1093/ijnp/pyac080, PMID: 36545813 PMC10032294

[12] Ballester PL Romano MT de Azevedo Cardoso T Hassel S Strother SC Kennedy SH. Brain age in mood and psychotic disorders: A systematic review and meta-analysis. Acta Psychiatrica Scandinavica. (2022) 145:42–55. doi: 10.1111/acps.13371, PMID: 34510423

[13] Rokicki J Wolfers T Nordhøy W Tesli N Quintana DS Alnæs D. Multimodal imaging improves brain age prediction and reveals distinct abnormalities in patients with psychiatric and neurological disorders. Hum Brain Mapp. (2021) 42:1714–26. doi: 10.1002/hbm.25323, PMID: 33340180 PMC7978139

[14] Huang J Ke P Chen X Li S Zhou J Xiong D. Multimodal magnetic resonance imaging reveals aberrant brain age trajectory during youth in schizophrenia patients. Front Aging Neurosci. (2022) 14:823502. doi: 10.3389/fnagi.2022.823502, PMID: 35309897 PMC8929292

[15] Demro C Shen C Hendrickson TJ Arend JL Disner SG Sponheim SR. Advanced brain-age in psychotic psychopathology: evidence for transdiagnostic neurodevelopmental origins. Front Aging Neurosci. (2022) 14:872867. doi: 10.3389/fnagi.2022.872867, PMID: 35527740 PMC9074783

[16] Niu X Zhang F Kounios J Liang H. Improved prediction of brain age using multimodal neuroimaging data. Hum Brain Mapp. (2020) 41:1626–43. doi: 10.1002/hbm.24899, PMID: 31837193 PMC7267976

[17] Zhu J-D Wu Y-F Tsai S-J Lin C-P Yang AC. Investigating brain aging trajectory deviations in different brain regions of individuals with schizophrenia using multimodal magnetic resonance imaging and brain-age prediction: A multicenter study. Trans Psychiatry. (2023) 13:82. doi: 10.1038/s41398-023-02379-5, PMID: 36882419 PMC9992684

[18] Jirsaraie RJ Kaufmann T Bashyam V Erus G Luby JL Westlye LT. Benchmarking the generalizability of brain age models: challenges posed by scanner variance and prediction bias. Hum Brain Mapp. (2023) 44:1118–28. doi: 10.1002/hbm.26144, PMID: 36346213 PMC9875922

[19] Jirsaraie RJ Gorelik AJ Gatavins MM Engemann DA Bogdan R Barch DM. A systematic review of multimodal brain age studies: uncovering a divergence between model accuracy and utility. Patterns. (2023) 4. doi: 10.1016/j.patter.2023.100712, PMID: 37123443 PMC10140612

[20] Dörfel RP Arenas-Gomez JM Fisher PM Ganz M Knudsen GM Svensson JE. Prediction of brain age using structural magnetic resonance imaging: A comparison of accuracy and test–retest reliability of publicly available software packages. Hum Brain Mapp. (2023) 44(17):6139–48. doi: 10.1002/hbm.26502, PMID: 37843020 PMC10619370

[21] Tetereva A Pat N. Brain age has limited utility as a biomarker for capturing fluid cognition in older individuals. Elife. (2024) 12:RP87297. doi: 10.7554/eLife.87297, PMID: 38869938 PMC11175613

[22] Kelly S Jahanshad N Zalesky A Kochunov P Agartz I Alloza C. Widespread white matter microstructural differences in schizophrenia across 4322 individuals: results from the enigma schizophrenia dti working group. Mol Psychiatry. (2018) 23:1261–9. doi: 10.1038/mp.2017.170, PMID: 29038599 PMC5984078

[23] Karlsgodt KH. Diffusion imaging of white matter in schizophrenia: progress and future directions. Biol Psychiatry. (2016) 1:209–17. doi: 10.1016/j.bpsc.2015.12.001, PMID: 27453952 PMC4955654

[24] Kuswanto CN Teh I Lee T-S Sim K. Diffusion tensor imaging findings of white matter changes in first episode schizophrenia: A systematic review. Clin Psychopharmacol Neurosci. (2012) 10:13. doi: 10.9758/cpn.2012.10.1.13, PMID: 23429992 PMC3569158

[25] Male AG Goudzwaard E Nakahara S Turner JA Calhoun VD Mueller BA. Structural white matter abnormalities in schizophrenia and associations with neurocognitive performance and symptom severity. Psychiatry Res. (2024) 342:111843. doi: 10.1016/j.pscychresns.2024.111843, PMID: 38896909

[26] Lener MS Wong E Tang CY Byne W Goldstein KE Blair NJ. White matter abnormalities in schizophrenia and schizotypal personality disorder. Schizophr Bull. (2015) 41:300–10. doi: 10.1093/schbul/sbu093, PMID: 24962608 PMC4266294

[27] Alkan E Davies G Evans SL. Cognitive impairment in schizophrenia: relationships with cortical thickness in fronto-temporal regions, and dissociability from symptom severity. NPJ Schizophr. (2021) 7:20. doi: 10.1038/s41537-021-00149-0, PMID: 33737508 PMC7973472

[28] Kay SR Fiszbein A Opler LA. The positive and negative syndrome scale (Panss) for schizophrenia. Schizophr Bull. (1987) 13:261–76. doi: 10.1093/schbul/13.2.261, PMID: 3616518

[29] Tesli N Bell C Hjell G Fischer-Vieler T Maximov II Richard G. The age of violence: mapping brain age in psychosis and psychopathy. Neuroimage: Clin. (2022) 36:103181. doi: 10.1016/j.nicl.2022.103181, PMID: 36088844 PMC9474919

[30] Drucker H Burges CJ Kaufman L Smola A Vapnik V. Support vector regression machines. Adv Neural Inf Process Syst. (1996) 9.

[31] Tipping ME. Sparse bayesian learning and the relevance vector machine. J Mach Learn Res. (2001) 1:211–44.

[32] Tibshirani R. Regression shrinkage and selection via the lasso. J R Stat Soc Ser B. (1996) 58:267–88. doi: 10.1111/j.2517-6161.1996.tb02080.x

[33] Rasmussen CE Williams CKI. Gaussian Processes for Machine Learning. Adaptive Computation and Machine Learning Series. Cambridge, MA: MIT Press. (2006). doi: 10.7551/mitpress/3206.001.0001

[34] Breiman L. Random forests. Mach Learn. (2001) 45:5–32. doi: 10.1023/A:1010933404324

[35] Tipping ME Bishop CM. Probabilistic principal component analysis. J R Stat Soc Ser B. (1999) 61:611–22. doi: 10.1111/1467-9868.00196

[36] Van Essen DC Smith SM Barch DM Behrens TE Yacoub E Ugurbil K. The wu-minn human connectome project: an overview. Neuroimage. (2013) 80:62–79. doi: 10.1016/j.neuroimage.2013.05.041, PMID: 23684880 PMC3724347

[37] Taylor JR Williams N Cusack R Auer T Shafto MA Dixon M. The cambridge centre for ageing and neuroscience (Cam-can) data repository: structural and functional mri, meg, and cognitive data from a cross-sectional adult lifespan sample. neuroimage. (2017) 144:262–9. doi: 10.1016/j.neuroimage.2015.09.018, PMID: 26375206 PMC5182075

[38] Liu W Wei D Chen Q Yang W Meng J Wu G. Longitudinal test-retest neuroimaging data from healthy young adults in southwest China. Sci Data. (2017) 4:1–9. doi: 10.1038/sdata.2017.17, PMID: 28195583 PMC5308199

[39] Zuo X-N Anderson JS Bellec P Birn RM Biswal BB Blautzik J. An open science resource for establishing reliability and reproducibility in functional connectomics. Sci Data. (2014) 1:1–13. doi: 10.1038/sdata.2014.49, PMID: 25977800 PMC4421932

[40] Aine C Bockholt HJ Bustillo JR Cañive JM Caprihan A Gasparovic C. Multimodal neuroimaging in schizophrenia: description and dissemination. Neuroinformatics. (2017) 15:343–64. doi: 10.1007/s12021-017-9338-9, PMID: 28812221 PMC5671541

[41] Gaser C Dahnke R Thompson PM Kurth F Luders E. Initiative asDN. Cat–a computational anatomy toolbox for the analysis of structural mri data. biorxiv. (2022) 13:giae049. doi: 10.1101/2022.06.11.495736 PMC11299546 39102518

[42] Yeh F-C Verstynen TD Wang Y Fernández-Miranda JC Tseng W-YI. Deterministic diffusion fiber tracking improved by quantitative anisotropy. PLoS One. (2013) 8:e80713. doi: 10.1371/journal.pone.0080713, PMID: 24348913 PMC3858183

[43] Dunås T Wåhlin A Nyberg L Boraxbekk C-J. Multimodal image analysis of apparent brain age identifies physical fitness as predictor of brain maintenance. Cereb Cortex. (2021) 31:3393–407. doi: 10.1093/cercor/bhab019, PMID: 33690853 PMC8196254

[44] Javitt DC. Glutamate and schizophrenia: phencyclidine, N-methyl-D-aspartate receptors, and dopamine–glutamate interactions. Int Rev Neurobiol. (2007) 78:69–108. doi: 10.1016/S0074-7742(06)78003-5, PMID: 17349858

[45] Jahanshad N Kochunov PV Sprooten E Mandl RC Nichols TE Almasy L. Multi-site genetic analysis of diffusion images and voxelwise heritability analysis: A pilot project of the enigma–dti working group. Neuroimage. (2013) 81:455–69. doi: 10.1016/j.neuroimage.2013.04.061, PMID: 23629049 PMC3729717

[46] Cole JH Ritchie SJ Bastin ME Hernández V Muñoz Maniega S Royle N. Brain age predicts mortality. Mol Psychiatry. (2018) 23:1385–92. doi: 10.1038/mp.2017.62, PMID: 28439103 PMC5984097

[47] Franke K Ziegler G Klöppel S Gaser C Initiative AsDN. Estimating the age of healthy subjects from T1-weighted mri scans using kernel methods: exploring the influence of various parameters. Neuroimage. (2010) 50:883–92. doi: 10.1016/j.neuroimage.2010.01.005, PMID: 20070949

[48] Baecker L Dafflon J Da Costa PF Garcia-Dias R Vieira S Scarpazza C. Brain age prediction: A comparison between machine learning models using region-and voxel-based morphometric data. Hum Brain Mapp. (2021) 42:2332–46. doi: 10.1002/hbm.25368, PMID: 33738883 PMC8090783

[49] Cole JH Franke K Cherbuin N. Quantification of the biological age of the brain using neuroimaging. Biomarkers Hum Aging. (2019), 293–328. doi: 10.1007/978-3-030-24970-0_19

[50] Valizadeh S Hänggi J Mérillat S Jäncke L. Age prediction on the basis of brain anatomical measures. Hum Brain Mapp. (2017) 38:997–1008. doi: 10.1002/hbm.23434, PMID: 27807912 PMC6866800

[51] Cole JH. Multimodality neuroimaging brain-age in uk biobank: relationship to biomedical, lifestyle, and cognitive factors. Neurobiol Aging. (2020) 92:34–42. doi: 10.1016/j.neurobiolaging.2020.03.014, PMID: 32380363 PMC7280786

[52] Ball G Kelly CE Beare R Seal ML. Individual variation underlying brain age estimates in typical development. Neuroimage. (2021) 235:118036. doi: 10.1016/j.neuroimage.2021.118036, PMID: 33838267

[53] Han J Kim SY Lee J Lee WH. Brain age prediction: A comparison between machine learning models using brain morphometric data. Sensors. (2022) 22:8077. doi: 10.3390/s22208077, PMID: 36298428 PMC9608785

[54] Lee WH. The choice of machine learning algorithms impacts the association between brain-predicted age difference and cognitive function. Mathematics. (2023) 11:1229. doi: 10.3390/math11051229

[55] Lombardi A Monaco A Donvito G Amoroso N Bellotti R Tangaro S. Brain age prediction with morphological features using deep neural networks: results from predictive analytic competition 2019. Front Psychiatry. (2021) 11:619629. doi: 10.3389/fpsyt.2020.619629, PMID: 33551880 PMC7854554

[56] Pedregosa F Varoquaux G Gramfort A Michel V Thirion B Grisel O. Scikit-learn: machine learning in python. J Mach Learn Res. (2011) 12:2825–30. doi: 10.48550/arXiv.1201.0490

[57] Engemann DA Kozynets O Sabbagh D Lemaître G Varoquaux G Liem F. Combining magnetoencephalography with magnetic resonance imaging enhances learning of surrogate-biomarkers. Elife. (2020) 9:e54055. doi: 10.7554/eLife.54055, PMID: 32423528 PMC7308092

[58] Liem F Varoquaux G Kynast J Beyer F Masouleh SK Huntenburg JM. Predicting brain-age from multimodal imaging data captures cognitive impairment. Neuroimage. (2017) 148:179–88. doi: 10.1016/j.neuroimage.2016.11.005, PMID: 27890805

[59] Liang H Zhang F Niu X. Investigating systematic bias in brain age estimation with application to post-traumatic stress disorders. Wiley Online Library. (2019) 40(11):1065–9471. doi: 10.1002/hbm.24588, PMID: 30924225 PMC6865701

[60] de Lange A-MG Cole JH. Commentary: correction procedures in brain-age prediction. NeuroImage. (2020) 26:102229. doi: 10.1016/j.nicl.2020.102229, PMID: 32120292 PMC7049655

[61] Zhang B Zhang S Feng J Zhang S. Age-level bias correction in brain age prediction. NeuroImage: Clin. (2023) 37:103319. doi: 10.1016/j.nicl.2023.103319, PMID: 36634514 PMC9860514

[62] Benjamini Y Hochberg Y. Controlling the false discovery rate: A practical and powerful approach to multiple testing. J R Stat Soc. (1995) 57:289–300. doi: 10.1111/j.2517-6161.1995.tb02031.x

[63] Nakagawa S Cuthill IC. Effect size, confidence interval and statistical significance: A practical guide for biologists. Biol Rev. (2007) 82:591–605. doi: 10.1111/j.1469-185X.2007.00027.x, PMID: 17944619

[64] Frangou S Modabbernia A Williams SC Papachristou E Doucet GE Agartz I. Cortical thickness across the lifespan: data from 17,075 healthy individuals aged 3–90 years. Hum Brain Mapp. (2022) 43:431–51. doi: 10.1002/hbm.25364, PMID: 33595143 PMC8675431

[65] Dima D Modabbernia A Papachristou E Doucet GE Agartz I Aghajani M. Subcortical volumes across the lifespan: data from 18,605 healthy individuals aged 3–90 years. Hum Brain Mapp. (2022) 43:452–69. doi: 10.1002/hbm.25320, PMID: 33570244 PMC8675429

[66] Joo SW Lee J Han J Kim M Kim Y Lee H. Disparities in accelerated brain aging in recent-onset and chronic schizophrenia. psychol Med. (2025) 55:e60. doi: 10.1017/S0033291725000285, PMID: 39988480 PMC12080660

[67] Kim W-S Heo D-W Maeng J Shen J Tsogt U Odkhuu S. Deep learning-based brain age prediction in patients with schizophrenia spectrum disorders. Schizophr Bull. (2024) 50:804–14. doi: 10.1093/schbul/sbad167, PMID: 38085061 PMC11283195

[68] Chen C-L Hwang TJ Tung Y-H Yang L-Y Hsu Y-C Liu CM. Detection of advanced brain aging in schizophrenia and its structural underpinning by using normative brain age metrics. NeuroImage: Clin. (2022) 34:103003. doi: 10.1016/j.nicl.2022.103003, PMID: 35413648 PMC9018160

[69] Mouches P Wilms M Rajashekar D Langner S Forkert ND. Multimodal biological brain age prediction using magnetic resonance imaging and angiography with the identification of predictive regions. Hum Brain Mapp. (2022) 43:2554–66. doi: 10.1002/hbm.25805, PMID: 35138012 PMC9057090

