# Supplementary material for: A multimodal ensemble stacking model improves brain age prediction and reveals associations with schizophrenia symptoms
Source: Front Psychiatry. 2025 Sep 4;16:1600479. doi: 10.3389/fpsyt.2025.1600479 (PMC12443690; doi:10.3389/fpsyt.2025.1600479)
Supplement: Supplementary file 1 [file DataSheet1.pdf]

Supplementary Material

1 Machine Learning Algorithms

1.1 Support Vector Regression

Support vector regression (SVR) is a machine learning algorithm well-suited for tasks like brain age prediction where we aim to model the relationship between neuroimaging features and chronological age. SVR is effective at handling high-dimensional data, which is a common characteristic of neuroimaging features extracted from sMRI and FA scans (1). While SVR can incorporate non-linear relationships through the use of kernel functions, this study specifically employed a linear kernel, which assumes a linear relationship between brain structure and age. This choice allows for a simple and interpretable model that serves as a baseline for comparison with more complex approaches, such as the proposed multimodal stacking model. In our implementation, SVR finds a linear function that minimizes prediction error while maintaining a flat, parsimonious representation, which helps prevent overfitting and supports generalizability to unseen internal test data. Although non-linear kernels may be explored in future work to capture more complex relationships, the linear kernel provides a foundational reference point for model evaluation.

1.2 Relevance Vector Regression

Similar to SVR, relevance vector regression (RVR) is a machine learning algorithm suitable for high-dimensional data, making it well-suited for brain age prediction using sMRI and FA features (2). However, RVR operates within a Bayesian framework, offering distinct advantages. Unlike SVR with its predefined cost parameter, RVR estimates hyperparameters and model complexity automatically during the learning process. This can be particularly beneficial for brain age prediction, where the optimal model complexity might not be readily apparent. Additionally, RVR inherently promotes sparsity, meaning it tends to select a smaller subset of relevant features to build the model. This can be advantageous for interpreting the model and potentially identifying the most influential neuroimaging features associated with brain age prediction. In our study, we employed RVR alongside other regression algorithms to compare its performance for brain age prediction and explore the potential benefits of its sparse representation for interpreting the relationship between neuroimaging features and brain age.

1.3 Least Absolute Shrinkage and Selection Operator Regression

Least absolute shrinkage and selection operator (Lasso) regression is a well-established technique for performing regularized linear regression (3-6). It applies an L1-norm penalty that shrinks the regression coefficients towards zero, potentially driving some coefficients to become exactly zero. This characteristic allows Lasso to perform feature selection by identifying and discarding irrelevant features that contribute little to the model's predictive power (7). This can be particularly advantageous in brain age prediction where a large number of features are extracted from neuroimaging data. In our study, we included Lasso alongside other algorithms to explore the

effectiveness of different regularization approaches for brain age prediction and to potentially gain insights into the most informative features for this task.

1.4 Gaussian Process Regression

Gaussian process regression (GPR) is a non-parametric model that offers flexibility in capturing complex relationships between neuroimaging features and chronological age (8), making it well-suited for brain age prediction tasks. Unlike traditional regression models that assume a fixed functional form, GPR learns the relationship directly from the data, using kernel functions to map similar data points to predictions. This allows GPR to model non-linear relationships between brain structure (e.g., sMRI and FA) and age. In addition to its predictive capabilities, GPR provides uncertainty estimates, which can indicate the model's confidence in its predictions. High uncertainty can highlight instances where the model encounters difficulties, such as outliers or data limitations. In this study, we used GPR to explore its ability to capture complex relationships in brain age prediction and provide useful uncertainty estimates.

1.5 Random Forest Regression

Random Forest (RF) regression is an ensemble learning method that utilizes multiple decision trees to predict brain age from sMRI and FA features (9). Each tree in the forest is trained on a random subset of features and data, which improves the model's robustness to outliers and reduces the risk of overfitting. The final brain age prediction is generated by averaging the outputs of all individual trees, leading to improved accuracy and stability compared to a single decision tree. RF regression is particularly effective for handling high-dimensional data, such as neuroimaging features, where a large number of variables are involved. Furthermore, RF is adept at modeling complex, non-linear relationships, which is crucial in brain age prediction, as the relationship between brain structure and age may not be linear. We employed RF regression due to its ability to handle diverse features, mitigate overfitting, and effectively model the complexities inherent in brain age prediction tasks.

Supplementary Table S1. Shapiro-Wilk normality test results for PANSS symptom scores and brainPAD values in the schizophrenia cohort.

Data	Shaprio-Wilk Test		
	W	p-value	H_0 Reject
Positive Scale	0.954	0.063	No
Negative Scale	0.957	0.081	No
General Psychopathology Scale	0.902	<0.001	Yes
PANSS Total	0.918	0.002	Yes
brainPAD (sMRI)	0.777	<0.001	Yes
brainPAD (FA)	0.353	<0.001	Yes
brainPAD (sMRI + FA)	0.790	<0.001	Yes

Supplementary Table S2. Pearson correlation coefficients between brainPAD values and PANSS symptom scores in schizophrenia patients, controlling for chronological age and sex.

Symptom Scale	sMRI			FA			sMRI + FA		
	r	*p*	FDR-*p*	r	*p*	FDR-*p*	r	*p*	FDR-*p*
Positive Scale	0.217	0.141	0.174	0.034	0.816	0.839	0.192	0.195	0.195
Negative Scale	0.201	0.174	0.174	-0.060	0.684	0.839	0.204	0.167	0.195
General Psychopathology Scale	0.424	0.002	0.011	-0.037	0.802	0.839	0.394	0.006	0.024
PANSS Total	0.372	0.009	0.019	-0.030	0.839	0.839	0.348	0.016	0.032

Supplementary Table S3. Effect sizes (Cohen's d) for age-corrected brainPAD values between healthy controls (HC) and patients with schizophrenia (SZ) in the COBRE sample, controlling for chronological age and sex.

Dataset used for age-bias correction	sMRI	FA	sMRI+FA
Internal Training Sample	0.583	0.297	0.734
COBRE HC Sample	0.739	0.523	0.769

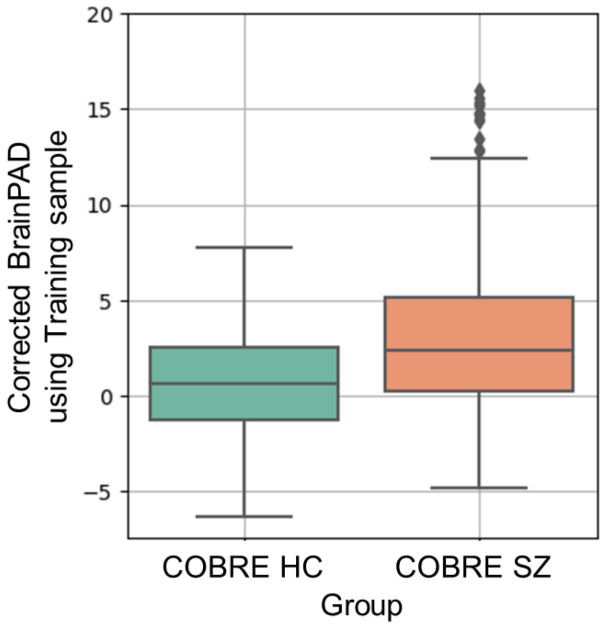

Supplementary Figure S1. Boxplots of age-corrected brainPAD for healthy controls (HC) and patients with schizophrenia (SZ) in the COBRE sample, using correction based on training sample.

2 Reference

1. Drucker H, Burges CJ, Kaufman L, Smola A, Vapnik V. Support Vector Regression Machines. *Advances in neural information processing systems* (1996) 9.

2. Tipping ME. Sparse Bayesian Learning and the Relevance Vector Machine. *Journal of machine learning research* (2001) 1(Jun):211-44.

3. Cole JH. Multimodality Neuroimaging Brain-Age in Uk Biobank: Relationship to Biomedical, Lifestyle, and Cognitive Factors. *Neurobiology of aging* (2020) 92:34-42.

4. Lee WH, Antoniades M, Schnack HG, Kahn RS, Frangou S. Brain Age Prediction in Schizophrenia: Does the Choice of Machine Learning Algorithm Matter? *Psychiatry Research: Neuroimaging* (2021) 310:111270.

5. Han J, Kim SY, Lee J, Lee WH. Brain Age Prediction: A Comparison between Machine Learning Models Using Brain Morphometric Data. *Sensors* (2022) 22(20):8077.

6. Lombardi A, Monaco A, Donvito G, Amoroso N, Bellotti R, Tangaro S. Brain Age Prediction with Morphological Features Using Deep Neural Networks: Results from Predictive Analytic Competition 2019. *Frontiers in Psychiatry* (2021) 11:619629.

7. Tibshirani R. Regression Shrinkage and Selection Via the Lasso. *Journal of the Royal Statistical Society Series B: Statistical Methodology* (1996) 58(1):267-88.

8. Williams CK, Rasmussen CE. *Gaussian Processes for Machine Learning*: MIT press Cambridge, MA (2006).

9. Breiman L. Random Forests. *Machine learning* (2001) 45:5-32.
